# Supplementary material for: Mesenchymal stromal cell apoptosis is required for their therapeutic function
Source: Nat Commun. 2021 Nov 11;12:6495. doi: 10.1038/s41467-021-26834-3 (PMC8586224; doi:10.1038/s41467-021-26834-3)
Supplement: Supplementary file 1 — Supplementary Information [file 41467_2021_26834_MOESM1_ESM.pdf]

SUPPLEMENTARY INFORMATION

Mesenchymal stromal cell apoptosis is required for their therapeutic function

Swee Heng Milon Pang[1], Joshua D'Rozario[1,2], Senora Mendonca[1], Tejasvini Bhuvan[1], Natalie Payne[3], Di Zheng[1], Assifa Hisana[1], Georgia Wallis[1], Adele Barugahare[4], David Powell[4], Jai Rautela[2], Nicholas D. Huntington[2], Grant Dewson[5], David C.S. Huang[5], Daniel H. D. Gray[5], Tracy S. P. Heng[1]*

[1] Department of Anatomy and Developmental Biology, Biomedicine Discovery Institute, Monash University, Clayton, VIC 3800, Australia. [2] Department of Biochemistry and Molecular Biology, Biomedicine Discovery Institute, Monash University, Clayton, VIC 3800, Australia. [3] Australian Regenerative Medicine Institute, Monash University, Clayton, VIC 3800, Australia. [4] Monash Bioinformatics Platform, Monash University, Clayton, VIC 3800, Australia. [5] The Walter and Eliza Hall Institute of Medical Research, Parkville, VIC 3052, Australia; Department of Medical Biology, University of Melbourne, Parkville, VIC 3010, Australia. These authors contribute equally: Joshua D'Rozario, Senora Mendonca. *Correspondence should be addressed to: Tracy.Heng@monash.edu

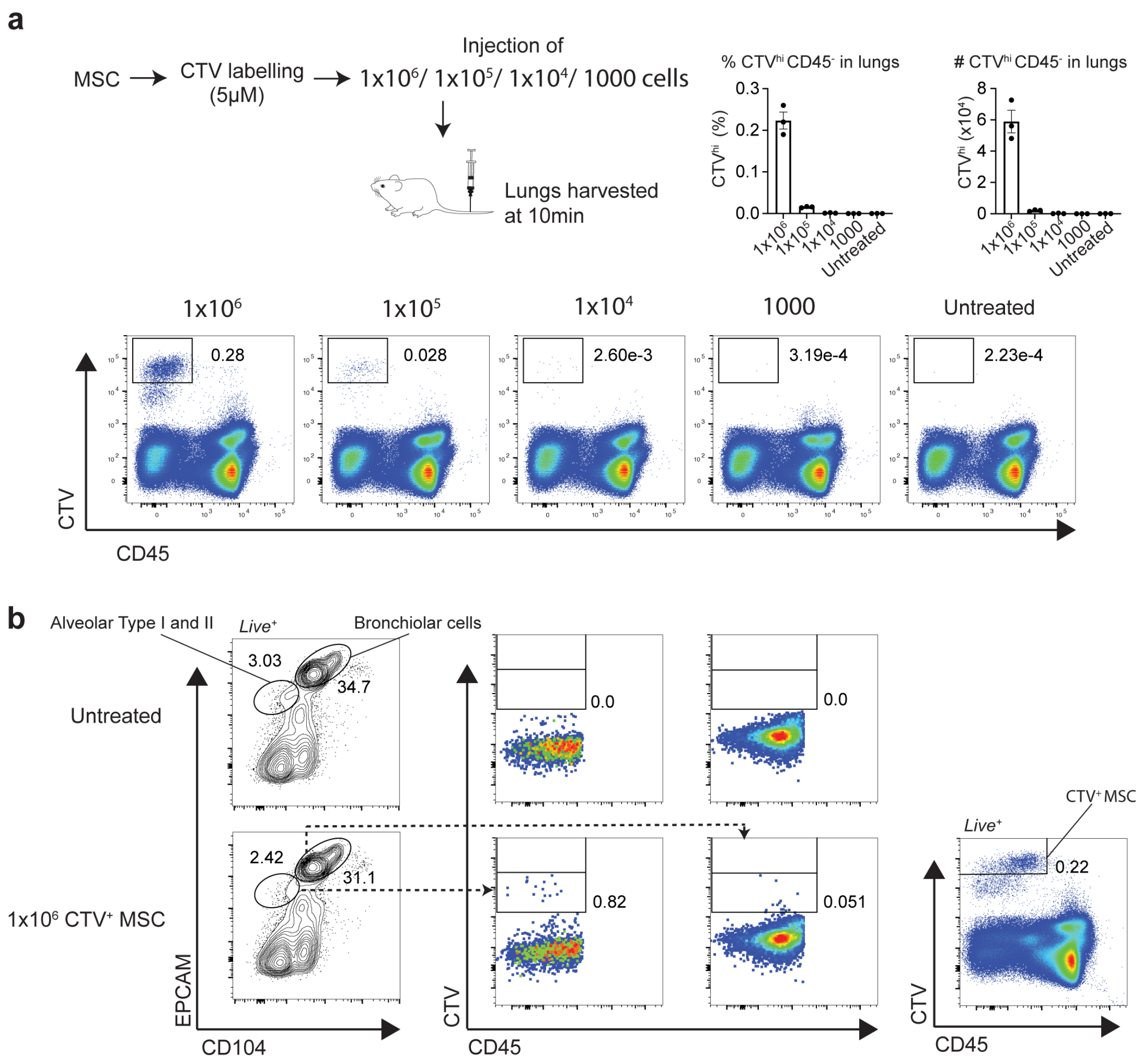

Supplementary Figure 1

Supplementary Figure 1. Detection of CTV-labelled MSC in the lungs after i.v. administration into BALB/c mice. a MSCs were labelled with CTV and i.v. injected at indicated cell numbers. MSCs were re-isolated from mouse lungs at 10 min. Top panel shows frequency and total number of $CTV^{hi}CD45^{-}$ population. Lower panel shows representative flow cytometric plots of $CTV^{hi}CD45^{-}$ population. Data expressed as mean $\pm$ SEM. Data representative of two independent experiments. *n* = 3 mice per group**. b** Left panels show representative flow cytometric plots of $CTV^{lo}CD45^{-}$ population that consists of alveolar type I and II, and bronchiolar cells that had engulfed injected MSCs. Right-most panel shows representative flow cytometric plot of CTV^{+} MSCs identified as $CTV^{hi}CD45^{-}$ population re-isolated from mouse lungs 10 min post-injection, for comparison of CTV signal. Data representative of two independent experiments. *n* = 3 mice per group. Source data are provided as a Source Data file.

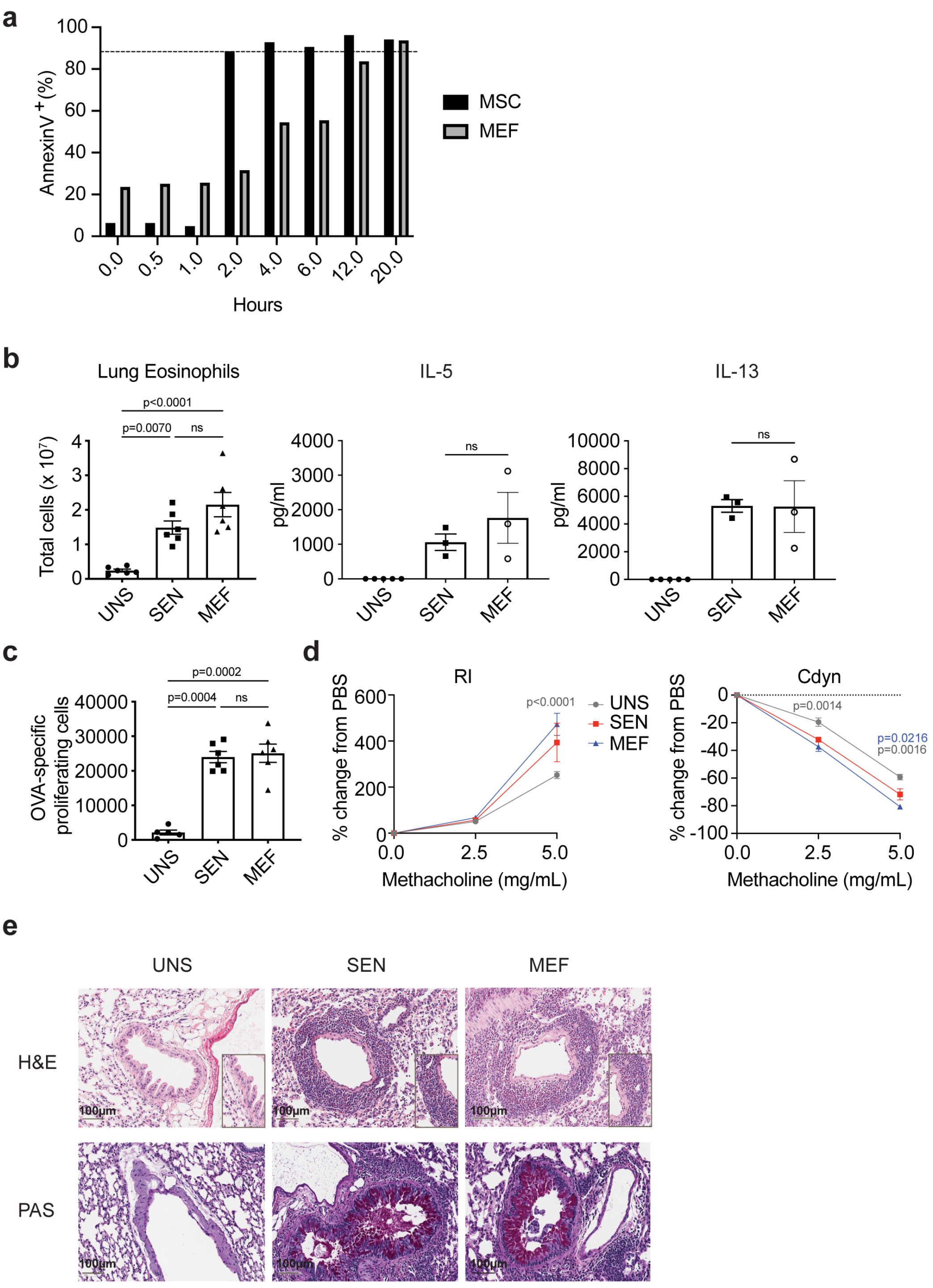

Supplementary Figure 2

Supplementary Figure 2. MEF treatment did not display immunosuppressive effects *in vivo*. **a** Frequency of AnnexinV$^+$ MSCs and MEFs following treatment with a combination of three BH3-mimetic drugs inhibiting MCL-1, BCL-2 and BCL-XL. MSCs were treated with 1.25uM of BH3-mimetics drugs, while MEFs were treated with 10uM. Data representative of two independent experiments. **b** OVA-sensitised mice received MEFs prior to OVA challenge. Number of eosinophils in the lungs (n = 6 mice per group), and IL-5 and IL-13 production after OVA stimulation of DLN cells (UNS n =5; SEN n = 3; MEF n = 3). Data expressed as mean $\pm$ SEM. Data representative of two independent experiments. p-values by one-way ANOVA (Tukey's post-hoc test). **c** OVA-specific DLN cell proliferation, measured by CFSE dilution. Data expressed as mean $\pm$ SEM (UNS n = 5; SEN n = 6; MEF n = 6). Data representative of two independent experiments. p-values by one-way ANOVA (Tukey's post-hoc test). **d** Measurement of RI and Cdyn in response to increasing doses of methacholine on Day 12. UNS = unsensitized mice; SEN = OVA-sensitized mice; MEF = OVA-sensitized mice that received MEFs. Data expressed mean $\pm$ SEM (UNS n = 5; SEN n = 4; MEF n = 6). p-values by two-way ANOVA (Tukey's post-hoc test) compared with SEN. **e** Lung sections were stained with H&E and PAS to analyse for pulmonary inflammation and mucus production respectively. Magnification 20x, scale bar = 100μm. Histological images were representative of 5 mice per group. Source data are provided as a Source Data file.

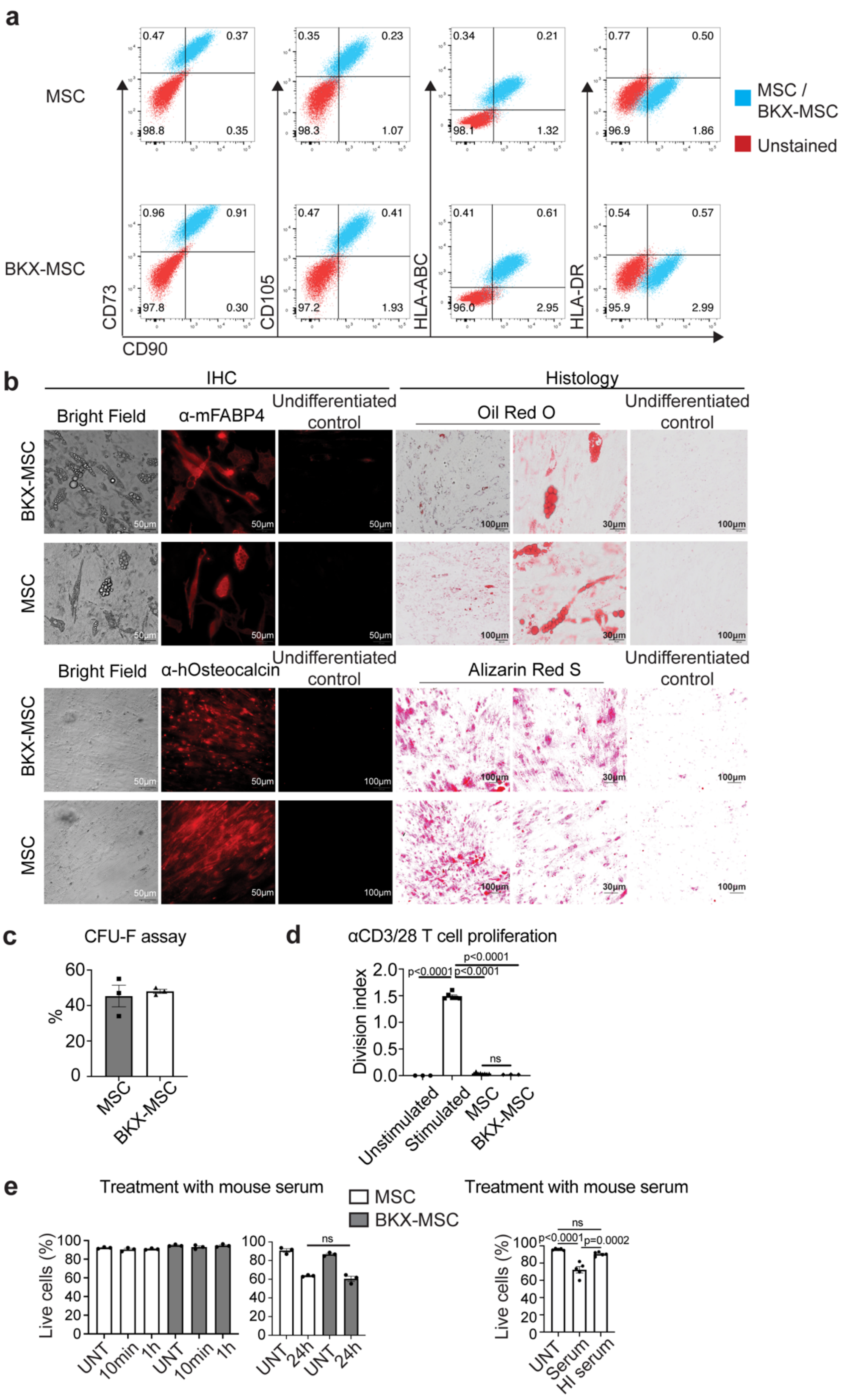

Supplementary Figure 3

Supplementary Figure 3. BKX-MSCs retain similar characteristics as MSCs. a Flow cytometric plots demonstrating that MSCs and BKX-MSCs express hCD73, hCD105, hCD90 and HLA-ABC but not HLA-DR. Data representative of two independent experiments. **b** BKX-MSCs showed similar differentiation capacity into mesenchymal lineages as MSCs when subjected to adipogenic and osteogenic differentiation assays. Top panels: immunohistochemistry (IHC) staining with α-mFABP4 (scale bar = 50μm) and Oil Red O (In this order, scale bar = 100μm; 30μm) staining for adipocytes. Lower panels: IHC staining with α-hOsteocalcin (scale bar = 50μm) and Alizarin Red S (In this order, scale bar = 100μm; 30μm) for osteoblasts. Undifferentiated MSCs (scale bar = 100μm) or BKX-MSCs controls (scale bar = 100μm) in normal culture medium were used as staining controls. Brightfield of MSCs and BKX-MSCs for IHC staining (scale bar = 50μm). Data representative over two independent experiments of which n = 3 samples per cell type. **c** MSCs and BKX-MSCs had similar clonogenic capacity when subjected to fibroblastic colony forming units (CFU-F) assay. Data representative over two independent experiments of which n = 3 samples per cell type. **d** MSCs and BKX-MSCs inhibited the proliferation of αCD3/CD28-stimulated CFSE-labelled purified T cells. Data expressed as mean ± SEM of two independent experiments (unstimulated n = 3; stimulated n = 6; MSC n = 8; BKX-MSC n =3). p-values by one-way ANOVA (Tukey's post-hoc test); ns, not significant. **e** Left panel shows frequency of live MSCs or BKX-MSCs following treatment with mouse serum at various timepoints. Data representative for two independent experiments of which n = 3 samples each timepoint. Right panel shows frequency of live MSCs following treatment with mouse serum and heat-inactivated (HI) mouse serum at 24 h. Data representative for two independent experiments of which n = 5 samples each treatment. Data expressed as mean ± SEM. p-values by

one-way ANOVA (Tukey's post-hoc test); ns, not significant. Source data are provided as a Source Data file.
