# Supplementary material for: Mesenchymal stromal cell apoptosis is required for their therapeutic function
Source: Nat Commun. 2021 Nov 11;12:6495. doi: 10.1038/s41467-021-26834-3 (PMC8586224; doi:10.1038/s41467-021-26834-3)
Supplement: Supplementary file 3 — Description of Additional Supplementary Files [file 41467_2021_26834_MOESM3_ESM.docx]

File name: Supplementary Movie 1

Description: Live cell imaging of CTG-labelled AMs (green) and pHrodo^TM^RED-labelled BH3-mimetic drug-treated MSCs (red) over 24 h, corresponding to snapshots in Fig. 5e. MSCs were engulfed by AMs shown here from 4 h to 10.5 h timepoints. Dead cells visualized with DRAQ7™ dye. Magnification 10x; Scale bar as indicated. Data representative of three experiments.

File name: Supplementary Movie 2

Description: Live cell imaging of CTG-labelled AMs (green) and pHrodo^TM^RED-labelled BH3-mimetic drug-treated BKX-MSCs (red) over 24 h, corresponding to snapshots in Fig. 5e. BKX-MSCs were not engulfed by AMs shown here from 4 h to 10.5 h timepoints. Dead cells visualized with DRAQ7™ dye. Magnification 10x; Scale bar as indicated. Data representative of three experiments.
