# Supplementary material for: Mesenchymal stromal cell apoptosis is required for their therapeutic function
Source: Nat Commun. 2021 Nov 11;12:6495. doi: 10.1038/s41467-021-26834-3 (PMC8586224; doi:10.1038/s41467-021-26834-3)
Supplement: Supplementary file 6 — Reporting Summary [file 41467_2021_26834_MOESM6_ESM.pdf]

nature research

Corresponding author(s): Tracy Heng

Last updated by author(s): Oct 15, 2021

Reporting Summary

Nature Research wishes to improve the reproducibility of the work that we publish. This form provides structure for consistency and transparency in reporting. For further information on Nature Research policies, see our Editorial Policies and the Editorial Policy Checklist.

Statistics

For all statistical analyses, confirm that the following items are present in the figure legend, table legend, main text, or Methods section.

n/a	Confirmed	
☐	☒	The exact sample size (*n*) for each experimental group/condition, given as a discrete number and unit of measurement
☐	☒	A statement on whether measurements were taken from distinct samples or whether the same sample was measured repeatedly
☐	☒	The statistical test(s) used AND whether they are one- or two-sided<br>*Only common tests should be described solely by name; describe more complex techniques in the Methods section.*
☐	☒	A description of all covariates tested
☐	☒	A description of any assumptions or corrections, such as tests of normality and adjustment for multiple comparisons
☐	☒	A full description of the statistical parameters including central tendency (e.g. means) or other basic estimates (e.g. regression coefficient) AND variation (e.g. standard deviation) or associated estimates of uncertainty (e.g. confidence intervals)
☐	☒	For null hypothesis testing, the test statistic (e.g. *F*, *t*, *r*) with confidence intervals, effect sizes, degrees of freedom and *P* value noted<br>*Give P values as exact values whenever suitable.*
☒	☐	For Bayesian analysis, information on the choice of priors and Markov chain Monte Carlo settings
☒	☐	For hierarchical and complex designs, identification of the appropriate level for tests and full reporting of outcomes
☒	☐	Estimates of effect sizes (e.g. Cohen's *d*, Pearson's *r*), indicating how they were calculated

Our web collection on statistics for biologists contains articles on many of the points above.

Software and code

Policy information about availability of computer code

Data collection	BD FACSDIva (BD Biosciences, v6.0) - to collect flow cytometric data<br>Living Image (Perkin Elmer, v3.2) - to collect in vivo bioluminescent images<br>Buxco FinePointe software (Data Sciences International) - to collect data for airway hyperresponsiveness
Data analysis	FlowJo (v10) - to analyse flow cytometric data collected from BD FACSDIVA, and to generate FACS plots and histograms<br>GraphPad Prism (v7) - to analyse and plot data for bar graphs and line graphs, and generate statistical comparison between data<br>Living Image software (Data Sciences International, v3.2) - to analyse and normalise in vivo bioluminescent images<br>FCAP Array software v3.0 (BD Biosciences) - to analyse flow cytometric data for cytokine profiiling<br>LEGENDplex Data Analysis Software v8 (Biolegend) - to analyse flow cytometric data for cytokine profiling<br>RNAsik (Monash University) - to process RNA-sequencing data<br>Degust (Monash University) - to analyse and visualise RNA-sequencing differential gene-expression data<br>GSEA v4.1.0 (Broad Institute) - to statistically analyse and interpret RNA expression data<br>Interferome v2.01 (Monash University) - to identify interferon-regulated genes from RNA-seq data<br>DAVID Functional Annotation v6.8 - to analyse pathway association of differential gene-expression data<br>Aperio ImageScope (Leica Biosystems, v12.4.3) - to analyse histology slides<br>ImageJ (imagej.nih.gov, 64-bit Java 1.8.0_172) - to analyse live cell imaging<br>Image Lab (Bio-Rad, v6.1) - to analyse western blot images

For manuscripts utilizing custom algorithms or software that are central to the research but not yet described in published literature, software must be made available to editors and reviewers. We strongly encourage code deposition in a community repository (e.g. GitHub). See the Nature Research guidelines for submitting code & software for further information.

Data

Policy information about availability of data

All manuscripts must include a data availability statement. This statement should provide the following information, where applicable:

- Accession codes, unique identifiers, or web links for publicly available datasets
- A list of figures that have associated raw data
- A description of any restrictions on data availability

The RNA-sequencing data generated in this study are available at NCBI GEO database under GSE156240 [https://www.ncbi.nlm.nih.gov/geo/query/acc.cgi?acc=GSE156240].

Field-specific reporting

Please select the one below that is the best fit for your research. If you are not sure, read the appropriate sections before making your selection.

☒ Life sciences ☐ Behavioural & social sciences ☐ Ecological, evolutionary & environmental sciences

For a reference copy of the document with all sections, see nature.com/documents/nr-reporting-summary-flat.pdf

Life sciences study design

All studies must disclose on these points even when the disclosure is negative.

Sample size	OVA-induced allergic asthma model (n = 6-8 mice per group); MOG-induced EAE model (n = 5-6 mice per group), antigen-specific proliferation and cytokine analyses - sample size of 6 mice per treatment group. Sample sizes were chosen to provide sufficient statistical power to detect differences.<br>RNA-sequencing of alveolar macrophages - each treatment group has 2 replicates, with each replicate FACSorted from BALF pooled from 5 mice.<br>Other assays in the study - sample size of 3 per treatment group.
Data exclusions	Data from positive control treatment animal group (OVA-sensitised group in asthma model) were excluded if the mice failed to display high airway resistance and low lung compliance during airway hyperresponsiveness assessment. These were then confirmed by flow cytometric analyses if the mice failed to present high number of eosinophils in the lungs. Similarly, antigen-specific proliferation and cytokine analyses correlate well with flow cytometric analyses; data that failed to give readings were excluded from further processing.
Replication	Two to three independent experimental replicates were performed. All attempts at replication were successful.
Randomization	Mice used in this study were randomly allocated to control or experimental groups. There was no bias in weight, size or behaviour of the animals. Passage 3-6 cells were used to prevent any biased results caused by passaging.
Blinding	In MOG-induced EAE model, the mice were scored blindly for clinical signs to avoid unconscious bias. In OVA-induced allergic asthma model, investigators were blinded to group allocation during lung histological preparation and analyses.

Reporting for specific materials, systems and methods

We require information from authors about some types of materials, experimental systems and methods used in many studies. Here, indicate whether each material, system or method listed is relevant to your study. If you are not sure if a list item applies to your research, read the appropriate section before selecting a response.

Materials & experimental systems

n/a	Involved in the study	
☐	☒	Antibodies
☐	☒	Eukaryotic cell lines
☒	☐	Palaeontology and archaeology
☐	☒	Animals and other organisms
☒	☐	Human research participants
☒	☐	Clinical data
☒	☐	Dual use research of concern

Methods

n/a	Involved in the study	
☒	☐	ChIP-seq
☐	☒	Flow cytometry
☒	☐	MRI-based neuroimaging

Antibodies

Antibodies used	Anti-mouse CD45 APC-Cy7 (Clone 30-F11) BD Cat# 557659<br>Anti-Active Caspase-3 PE (Clone C92-605) BD Cat# 550821<br>Annexin V Recombinant FITC BD Cat# 556419

	Anti-mouse CD11b BV650 (Clone M1/70) BD Cat# 563402<br>Anti-mouse CD45 FITC (Clone 30-F11) BD Cat# 553080<br>Anti-mouse CD11c BV421 (Clone N418) BD Cat# 565451<br>Anti-mouse Ly-6G A700 (Clone 1A8) BD Cat# 561236<br>Anti-mouse Ly-6C BV711 (Clone HK1.4) Biolegend Cat# 128037<br>Anti-mouse CD170 (Siglec F) SB780 (Clone 1RNM44N) ThermoFisher Scientific Cat# 78-1702-82<br>Anti-mouse CD64 PE-Cy7 (Clone X54-5/7.1) BIolegend Cat# 139314<br>Anti-mouse I-A/I-E BV510 (Clone M5/114.15.2) Biolegend Cat# 107636<br>Anti-mouse CD24 PerCP-Cy5.5 (Clone M1/69) Biolegend Cat# 101824<br>Anti-mouse CD16/32 Purified (Clone 2.4G2) Walter and Eliza Hall Institute; lab-made<br>Anti-BAX (7D10) Walter and Eliza Hall Institute; lab-made<br>Anti-BAK (aa23-32) Sigma-Aldrich<br>GAPDH (14C10) Rabbit mAb Cell Signaling Technology Cat# 2118<br>Anti-mouse CD4 PE-Cy7 (Clone RM4-5) ThermoFisher Scientific Cat# 25-0042-82<br>Anti-mouse CD8 APC (Clone 53-6.7) BD Cat# 553035<br>Anti-mouse CD3e Purified NA/LE (Clone 145-2C11) BD Cat# 553057<br>Anti-mouse CD28 Purified NA/LE (Clone 37.51) BD Cat# 553294<br>Anti-CD11b Biotin (Clone M1/70) BD Cat# 557395<br>Anti-mouse CD11c Biotin (Clone HL3) BD Cat# 553800<br>Anti-mouse CD19 Biotin (Clone 1D3) BD Cat# 553784<br>Anti-mouse CD45R/B220 Biotin (Clone Ra3-6B2) BD Cat# 553085<br>Anti-mouse NK1.1 Biotin (Clone PK136) BD Cat# 553163<br>CD326 (EpCAM) Monoclonal Antibody (Clone G8.8) Biotin ThermoFisher Scientific Cat# 13-5791-82<br>Anti-mouse MHC Class II (I-A/I-E) (Clone M5/114.15.2) Biotin ThermoFisher Scientific Cat# 36-5321-85<br>Anti-mouse TER-119/Erythroid Cells (Clone TER-119) Biotin BD Cat# 553672<br>Anti-human CD73 (Clone AD2) PE BD Cat# 550257<br>Anti-human CD90 (Clone 5E10) PE-Cy7 BD Cat# 561558<br>Anti-human CD105 (Clone 266) APC BD Cat# 561443<br>Anti-human HLA-ABC (Clone G46-2.6) FITC BD Cat# 555552<br>Anti-human HLA-DR (Clone G46-6) V450 BD Cat# 561359<br>Anti-mouse CD31 APC-Cy7 (Clone MEC13.3) Biolegend Cat# 102534<br>Anti-mouse CD104 FITC (Clone 346-11A) Biolegend Cat# 123606<br>Anti-mouse CD326/EpCAM PE-Cy7 (Clone G8.8) Biolegend Cat# 118216<br>Anti-mouse CD3e (Clone 145-2C11) Purified NA/LE BD Cat# 567114<br>Anti-mouse CD28 (Clone 37.51) Purified NA/LE BD Cat# 567110<br>Anti-mouse CD115 biotin (Clone AFS98) Biolegend Cat# 135508<br>Streptavidin PE-Cy7 Biolegend Cat# 405206<br>Streptavidin APC-Cy7 Biolegend Cat# 405208<br>Streptavidin BV510 Biolegend Cat# 405234
Validation	Each antibody used in this study was tested and titrated using cells from thymus, spleen, bone marrow or lungs of mice, or human cell lines. Each antibody was tested on cells at various dilutions (1:25, 1:50, 1:100. 1:200, 1:400, 1:800 and 1:1600) to determine the right fluorescence intensity. E.g. CD45 antibody was tested on lung cell suspension at various concentrations to determine the right fluorescence intensity as shown in Fig. 1b. In some cases, thymocytes were treated with 1uM Dexamethasone for 24h and used to titrate Caspase-3 and Annexin V antibodies.<br><br>Dilution of each antibody varies each time with different batch lots. Each antibody was tested first before any experiment.

Eukaryotic cell lines

Policy information about cell lines

Cell line source(s)	Human bone marrow-derived mesenchymal stromal cells (Tulane Centre for Gene Therapy, Tulane University, New Orleans, LA)<br>Human adipose-derived and umbilical cord-derived mesenchymal stromal cells (Monash University)<br>Mouse embryonic fibroblasts (MEFs) derived from C56Bl/6 mice (Walter and Eliza Hall Institute)<br>HeLa cell lines were from Walter and Eliza Hall Institute
Authentication	The cells have been rigorously tested to meet the criteria set by the International Society for Cell and Gene Therapy for the definition of multipotent mesenchymal stromal cells (MSCs). MSCs must be plastic-adherent when maintained in standard culture conditions, express CD105, CD73, CD90 and HLA-ABC, and be able to differentiate into osteoblasts and adipocytes in vitro (Supplementary Fig. 3).
Mycoplasma contamination	All cells have been tested negative for mycoplasma.
Commonly misidentified lines<br>(See ICLAC register)	HeLa cell lines

Animals and other organisms

Policy information about studies involving animals; ARRIVE guidelines recommended for reporting animal research

Laboratory animals	BALB/c and C57BL/6; female; 7- to 9- week old (Monash Animal Services)<br>NOD/SCID/Il2rgc−/− (NSG); female; 6- to 12- week old (Walter and Eliza Hall Institute)<br>BALB/c NOD.sirpa Rag2−/− Il2rgc−/− (BRGS); female; 6- to 12- week old (Walter and Eliza Hall Institute)<br>All animals were maintained under specific pathogen-free conditions. All experiments were conducted in accordance with the guidelines of the Australian Code of Practice for the Care and Use of Animals for Scientific Purposes and approved by the Animal Ethics committees.
Wild animals	The study did not involve wild animals.
Field-collected samples	The study did not involve samples collected from the field.
Ethics oversight	Female 7- to 9-week old BALB/c and C57BL/6 mice were obtained from Monash Animal Services and maintained under specific pathogen-free conditions at the Monash University Animal Research Laboratories. All animal experiments were conducted in accordance with the guidelines of the Australian Code of Practice for the Care and Use of Animals for Scientific Purposes and approved by the Monash University Animal Ethics committee (MARP/2016/160).

Note that full information on the approval of the study protocol must also be provided in the manuscript.

Flow Cytometry

Plots

Confirm that:

- [x] The axis labels state the marker and fluorochrome used (e.g. CD4-FITC).
- [x] The axis scales are clearly visible. Include numbers along axes only for bottom left plot of group (a 'group' is an analysis of identical markers).
- [x] All plots are contour plots with outliers or pseudocolor plots.
- [x] A numerical value for number of cells or percentage (with statistics) is provided.

Methodology

Sample preparation	Lungs were digested by DNAse I and Collagenase Type 1 as specified in the Methods. Spleens and lymph nodes were passed through cell strainers (70uM) to make single cell suspension.
Instrument	FACS Canto analyser, FACS LSRII analyser, FACS Fortessa analyser, BD Influx sorter
Software	FACS DIVA (v6.0), Flowjo (v10)
Cell population abundance	Approximately 1 million neutrophils, alveolar macrophages, and monocytes could be obtained from the lungs of 6 mice (untreated or MSC-treated OVA-sensitised mice), or 10 animals (OVA-sensitised animals). The purity of each population was confirmed post-sort to be >90%. Viability and cell counts were again confirmed by haemocytometer cell counting with trypan blue exclusion.
Gating strategy	Strategy for gating individual myeloid populations in the lung is provided in Figure 5a.

- [x] Tick this box to confirm that a figure exemplifying the gating strategy is provided in the Supplementary Information.
